# Clinico-epidemiological survey of feline parvovirus circulating in three Egyptian provinces from 2020 to 2021

**DOI:** 10.1007/s00705-023-05751-4

**Published:** 2023-03-30

**Authors:** Mohamed M.M. Abdel-Baky, Khaled A.S. El-Khabaz, Abdelbaset E. Abdelbaset, Maha I. Hamed

**Affiliations:** Assiut University Faculty of Veterinary Medicine, Assiut, Egypt

## Abstract

Feline parvovirus infection, caused by feline parvovirus and canine parvovirus 2, is a highly contagious, life-threatening disease affecting cats. The available epidemiological data on parvovirus infection in cats in Egypt is limited. Therefore, the aim of the current study was to provide data concerning the epidemiological profile of cats infected with parvovirus, including the prevalence of parvovirus infection in cats in three Egyptian provinces (Sohag, Assiut, and Cairo) and the associated risk factors. Using rapid antigen tests of fecal samples and conventional PCR, the overall prevalence of parvovirus infection in cats was found to be 35% (35/100) and 43% (43/100), respectively. Anorexia, bloody diarrhea, severe dehydration, hypothermia, and vomiting were the most common clinical findings significantly associated with parvovirus-infected cats. The geographical location (Sohag) and the season (winter) were both statistically significant risk factors for parvovirus infection. These findings indicate that parvoviruses are circulating in different regions of Egypt. Our study provides baseline epidemiological data for future preventive and control measures against parvovirus infection, as well as highlighting the need for future genomic surveillance studies involving a large study population from various parts of Egypt in order to better shape the epidemiological picture of parvovirus infection.

## Introduction

Feline panleukopenia (FPL)/feline parvovirus (FPV) infection is a highly contagious, life-threatening disease affecting all members of the family Felidae, typically characterized by acute enteritis, severe dehydration, vomiting, and sepsis as a result of lymphoid depletion and pancytopenia [1–3]. Parvovirus infection in cats is commonly associated with FPV. However, it can also be caused by variants of canine parvovirus 2 (CPV-2), specifically CPV-2a, b, and c [3]. Collectively, FPV and CPV-2 are included within the species *Carnivore protoparvovirus 1* (family *Parvoviridae*) and are found in a variety of carnivore and feline species [4–6].

Parvovirus infection occurs most frequently in young unvaccinated or incompletely vaccinated kittens [7, 8]. Importantly, even with treatment, the mortality rate of parvovirus infection ranges from 25% to 100%, depending on the severity of clinical signs [4]. Marked leucopenia or thrombocytopenia, as well as hypokalemia or hypoalbuminemia, are indicators of a poor prognosis [7, 8].

Despite the danger of parvovirus infection to cats and the growing interest in owning cats as pets, the epidemiological status of parvovirus infection is still unknown in most Egyptian localities. The only previous study of the prevalence of FPV in Egypt examined 165 diseased cats and found a prevalence of 40% and 45%, using ELISA and PCR, respectively [9]. Therefore, the aim of this study was to provide data concerning the epidemiological profile of cats infected with parvoviruses (FPV/CPV-2) in Egypt, including the prevalence of parvovirus infection among cats and the associated risk factors.

## Materials and methods

### Ethical approval

All procedures were performed following the ethical standards of the institutional ethics committee of Assiut University. All cat owners were informed about the objectives of the study and methodology, voluntary participation, and confidentiality of personal information. All cats were handled in compliance with Assiut University's regulations for animal research, and samples were collected with the owners' consent. The study was approved by the institutional ethics committee of Assiut University.

### Study area

The current study was conducted in three Egyptian provinces: Cairo, Assiut, and Sohag, from November 2020 to December 2021. Cats were admitted to the Imbaba Veterinary Hospital and Pets Care Clinic (Cairo), Smile Pet Clinic (Assiut), and Vet Pet Clinic (Sohag).

### Cats

This study included a total of 100 cats of different ages, sexes, and breeds. Only cats exhibiting one or more clinical signs of parvovirus infection (lethargy, anorexia, vomiting, and/or bloody diarrhea) were included.

### Data collection and clinical examination

Data obtained from each cat owner included the age (1-6 months, >6-12 months, or >12 months), sex (male or female), breed (Persian, Siamese, Egyptian Mau, or mixed breed), location (Sohag, Assiut, or Cairo), vaccination status, and living conditions (indoors or outdoors) of the cat. Clinical signs such as anorexia, lethargy, vomiting, diarrhea, and mucoid or bloody diarrhea were also recorded. All cats were physically examined on admission as described by Gaskell et al. [10]. Physical examination included body temperature measurement, visual examination of mucous membranes, and checking for signs of respiratory illness, digestive disturbances, or dehydration.

### Sampling

Stool samples were collected directly from the rectum using fecal swabs [11]. Each swab was submerged in 400 μl of sterile saline, and the suspension was divided into two parts; one was used immediately for rapid antigen tests, and the other was stored at -20 °C for molecular detection.

### Screening of cats for the presence of FPV and CPV-2 antigen

SNAP Parvovirus Antigen Test Kits, including SNAP Parvo (IDEXX Laboratories, USA) and VDRG ®CPV Ag Rapid Kit (Medain, Korea), which are based on ELISA and immunochromatographic technology, respectively, were used according to the manufacturer's instructions to screen for parvovirus infection in cats.

### Molecular detection

DNA was extracted from all fecal samples using a QIAamp Fast DNA Stool Mini Kit (QIAGEN, Germany) according to the manufacturer's instructions. Molecular screening was carried out by PCR using primers (Sigma-Aldrich) designed by Stephen Dunham for detection of the FPV VP-2 gene (felVP2-3820F, 5-TTGARGCRTCTACACAAGGG-3′; VP2-4247R, 5′-TGGTGCATTTACATGAAGTCTTGG-3′) [12]. The PCR reactions were carried out in a total volume of 25 µl, using the following PCR cycling conditions: initial denaturation at 95°C for 1 minute; 40 cycles of denaturation for 15 seconds at 95°C, primer annealing at 60°C for 15 seconds, and extension at 72°C for 15 seconds; and a final extension for 2 minutes at 72°C. The PCR products were visualized using 1.5% agarose gel electrophoresis and a UV transilluminator.

### Statistical analysis

To measure the impact of each factor individually on the prevalence of the disease in cats (i.e., age, sex, breed, season, locality, vaccination status, and lifestyle) and the association of clinical signs with the occurrence of the disease (i.e., temperature, activity, appetite, vomiting, diarrhea, and dehydration), relative risk was calculated and chi-square tests were performed using SPSS statistics software (IBM Corp, USA, Version 29). A probability value (*P*-value) less than 0.05 was considered statistically significant when comparing two groups, but Bonferroni correction of the *p*-value using the formula a_new_ = a_original_ /n was used when comparing more than two groups. *P* < 0.0167 was considered statistically significant when comparing three groups, while *P* < 0.0125 was considered statistically significant when comparing four groups.

## Results

Using rapid antigen tests and conventional PCR, the overall prevalence of parvovirus infection in cats was found to be 35% (35/100) and 43% (43/100), respectively. The size of the PCR product amplified from the FPV VP-2 gene was 428 base pairs, which was similar to that of the positive control.

Among the parvovirus PCR-positive cats, 86.05% (37/43) were lethargic, compared to 68.42% (39/57) of parvovirus-negative cats; 88.37% (38/43) had anorexia, compared to 70.2% (40/57) of negative cats; 11.63% (5/6) had hypothermia, compared to 1.76% (1/6) of negative cats; 74.4% (32/43) showed persistent vomiting, compared to 49.1% (28/57) of negative cats; 58.14% (25/43) had mucoid to bloody diarrhea, compared to 57.9% (33/57) of negative cats; and 30.23% (13/43) showed severe dehydration, compared to 10.53% (6/57) of negative cats (Table 1). There was a significant association between PCR test results and clinical signs in cats (Table 1). Cats with anorexia, bloody diarrhea, severe dehydration, hypothermia, and vomiting had a significantly higher prevalence of parvovirus infection than cats with normal appetite, mucoid diarrhea, no dehydration, normal body temperature, and absence of vomiting (Table 1).

**Table 1:** Association of clinical signs of parvovirus infection with rapid test and PCR results in the examined cats

Factor	No. tested	Rapid test		*P*-value	PCR test		*P*-value
		Positive n (%)	Negative n (%)		Positive n (%)	Negative n (%)	
Activity							
Normal	24	4 (11.43)	20 (30.77)	0.0480	6 (13.95)	18 (31.58)	0.0579
Lethargic	76	31 (88.57)	45 (69.23)		37 (86.05)	39 (68.42)	
Total	100	35 (100)	65 (100)	-	43 (100)	57 (100)	-
Appetite							
Normal	22	3 (8.57)	19 (29.23)	0.0220	5 (11.63)	17 (29.8)	0.0495
Anorexic	78	32 (91.43)	46 (70.77)		38 (88.37)	40 (70.2)	
Total	100	35 (100)	65 (100)	-	43 (100)	57 (100)	-
Fecal consistency							
Non-diarrheic	42	14 (40)	28 (43.1)	0.8336	18 (41.86)	24 (42.1)	0.9999
Diarrheic	58	21 (60)	37 (56.9)		25 (58.14)	33 (57.9)	
Total	100	35 (100)	65 (100)	-	43 (100)	57 (100)	-
Diarrheic form							
Mucoid	26	6 (28.6)	20 (54.1)	0.0986	7 (28)	19 (57.6)	0.0341
Bloody	32	15 (71.4)	17 (45.9)		18 (72)	14 (42.4)	
Total	58	21 (100)	37 (100)	-	25 (100)	33 (100)	-
Dehydration							
None	44	6 (17.14)	38 (58.5)	R	13 (30.23)	31 (54.39)	R
Mild	15	7 (20)	8 (12.3)	0.013	7 (16.28)	8 (14.03)	0.344
Moderate	22	8 (22.86)	14 (21.5)	0.054	10 (23.26)	12 (21.05)	0.274
Severe	19	14 (40)	5 (7.7)	<0.0001	13 (30.23)	6 (10.53)	0.006
Total	100	35 (100)	65 (100)	-	43 (100)	57 (100)	-
Temperature							
Normothermic	35	7 (20)	28 (43.1)	R	9 (20.93)	26 (45.61)	R
Hyperthermic	59	23 (65.7)	36 (55.4)	0.069	29 (67.44)	30 (52.63)	0.031
Hypothermic	6	5 (14.3)	1 (1.5)	0.005	5 (11.63)	1 (1.76)	0.013
Total	100	35 (100)	65 (100)	-	43 (100)	57 (100)	-
Vomiting							
Present	60	30 (85.7)	30 (46.15)	0.0001	32 (74.4)	28 (49.1)	0.0135
Absent	40	5 (14.3)	35 (53.85)		11 (25.6)	29 (50.9)	
Total	100	35 (100)	65 (100)	-	43 (100)	57 (100)	-

R (reference)

Bold indicates statistical significance

Among the risk factors analyzed, the geographic location and the season were statistically significant factors associated with parvovirus infection (Table 2). Cats from Sohag had a significantly higher prevalence rate (62.5%) than cats from Assiut and Cairo (27.3%, and 40%, respectively). Furthermore, cats sampled during the winter (54.5%) were significantly more frequently positive for parvovirus than cats sampled during the spring (46.1%), summer (15.8%), and autumn (41.7%). On the other hand, there was no significant relationship between the various risk factors analyzed (age, sex, breed, vaccination status, and lifestyle) and infection rate among the cats studied (Table 2).

**Table 2:** Risk factors associated with parvovirus prevalence among the examined cats

Factor	No. tested	Rapid test			PCR test		
		Positive n (%)	Negative n (%)	*P*-value	Positive n (%)	Negative n (%)	*P*-value
Age							
1-6 months	48	19 (39.6)	29 (60.4)	0.163	24 (50)	24 (50)	0.296
> 6-12 months	32	12 (37.5)	20 (62.5)	0.228	12 (37.5)	20 (62.5)	1.00
>12 months	20	4 (20)	16 (80)	R	7 (35)	13 (65)	R
Total	100	35	65	-	43	57	-
Sex							
Male	48	20 (41.7)	28 (58.3)	0.2113	24 (50)	24 (50)	0.2257
Female	52	15 (28.9)	37 (71.1)		19 (36.5)	33 (63.5)	
Total	100	35	65	-	43	57	
Breed							
Persian	66	22 (33.3)	44 (66.7)	0.780	27 (40.9)	39 (59.1)	0.797
Siamese	7	4 (57.1)	3 (42.9)	0.188	5 (71.4)	2 (28.6)	0.190
Egyptian Mau	8	4 (50)	4 (50)	0.375	4 (50)	4 (50)	0.675
Mixed	19	5 (26.3)	14 (73.7)	R	7 (36.8)	12 (63.2)	R
Total	100	35	65	-	43	57	-
Season							
Spring	13	8 (61.5)	5 (38.5)	0.039	6 (46.1)	7 (53.9)	0.109
Summer	19	0	19 (100)	-	3 (15.8)	16 (84.2)	R
Autumn	24	6 (25)	18 (75)	R	10 (41.7)	14 (58.3)	0.098
Winter	44	21 (47.7)	23 (52.3)	0.077	24 (54.5)	20 (45.5)	0.005
Total	100	35	65	-	43	57	-
Area							
Assiut	33	8 (24.2)	25 (75.8)	R	9 (27.3)	24 (72.7)	R
Sohag	32	18 (56.3)	14 (43.7)	0.012	20 (62.5)	12 (37.5)	0.006
Cairo	35	9 (25.7)	26 (74.3)	1.00	14 (40)	21 (60)	0.312
Total	100	35	65	-	43	57	-
Vaccination							
Yes	9	1 (11.1)	8 (88.9)	0.155	1 (11.1)	8 (88.9)	0.0738
No	91	34 (37.4)	57 (62.6)		42 (46.1)	49 (53.9)	
Total	100	35	65	-	43	57	-
In/outdoor							
Indoor	80	30 (37.5)	50 (62.5)	0.4324	38 (47.5)	42 (52.5)	0.0815
Outdoor	20	5 (25)	15 (75)		5 (25)	15 (75)	
Total	100	35	65	-	43	57	-

R (reference)

Bold indicates statistical significance

## Discussion

Feline parvovirus infection is a common disease that causes diarrhea in cats and can be life-threatening in severe cases. This study demonstrates that anorexia, bloody diarrhea, severe dehydration, hypothermia, and vomiting are the common clinical signs that are significantly associated with parvovirus infection in domestic cats in Egypt. These findings are similar to those of previous studies of FPV infection described by Mosallanejad et al. [13] and Miranda et al. [14]. Not all cats infected with FPV display clinical signs, and the disease severity varies with immune status, age, and concurrent infections [7].

Our survey showed that 35 cats were positive for parvovirus infection when using the rapid antigen test, while conventional PCR identified 43 positive cases. This difference may be attributed to false-negative results obtained when using fecal antigen tests. Due to its sensitivity and specificity, PCR is more reliable for detection of parvovirus in fecal samples [15, 16]. Notably, three out of the 35 positive samples (as determined by the rapid test) were found to be negative when tested by PCR. One of these three animals had recently been vaccinated. A similar result was also reported by Abd-Eldaim et al. [17], who also had one case in which the sample tested positive for parvovirus infection by the rapid test, but negative by PCR. False-positive results can be obtained by both the rapid test and PCR after vaccination because the virus is shed in feces [18], and therefore, the reason for the discrepancy between the results of the two tests in this case is still unknown.

False-positive results can occur in recently FPV-vaccinated kittens (within 14 days of vaccination) with modified live vaccines [19].

Furthermore, rapid assays can only detect parvoviruses in feces for up to 48 hours after infection, and their sensitivity and specificity vary among the different rapid tests and stages of infection [17, 20]**.**

The overall prevalence of parvovirus infection among the examined cats was 43% when using PCR. This high prevalence rate could be attributed to environmental contamination with the virus (FPV or CPV) or the lack of vaccination against FPV infection (91% of the examined cats were unvaccinated), since unvaccinated cats are more vulnerable to infection [21, 22]. Additionally, infected homeless cats, which roam about in search of food, contribute to disease transmission [23, 24]. Similar rates of FPV prevalence in Giza, Egypt, were reported by Awad et al. [9], who found a prevalence of FPV infection of 45% among 165 diseased cats, using PCR. In contrast to our findings, a lower prevalence of parvovirus (either FPV or CPV) infection, 8.15% of fecal samples obtained from 1719 apparently healthy cats, was found in the United Kingdom, using PCR, whereas a higher prevalence of parvovirus infection, 72.7% of blood samples obtained from 11 cats, was found in Indonesia, also using PCR [25]. These divergent results might be explained by differences in the vaccination rates between countries, differences in natural exposure to the virus, the study populations chosen, the use of different diagnostic tests, housing conditions, and the immune status of the cats [7].

Regarding the prevalence of the disease in different Egyptian provinces, the significantly higher infection rate in Sohag province may be attributed to a lower rate of vaccination among the cats tested in Sohag than in Assiut and Cairo.

The prevalence of parvovirus infection was found to be inversely proportional to age, without a significant difference, which is consistent with the results reported by Kim et al. [26], who observed a higher prevalence of parvovirus infection in kittens (less than one year old) than adult cats (more than one year old). Young kittens are more vulnerable to parvovirus infection because they lack antibody protection [8]. Furthermore, early weaning, an improper practice of some cat breeders to reduce breeding costs, results in a gradual decline in maternally derived antibody titers [27]. On the other hand, older cats are protected either through subclinical infection or previous vaccination [8].

The prevalence rate of parvovirus infection in relation to sex and breed of cat showed no significant difference. These findings are consistent with previous studies that found no significant difference in disease prevalence based on breed or gender [11, 13, 21, 23].

In our study, cats were significantly more frequently infected with parvovirus in the winter season. A similar seasonality of parvovirus infection among cats has been reported in New Zealand and Australia, where case numbers peaked from December to May [28]. In contrast, outbreaks commonly occur during July, August, and September in the United States due to admission of kittens that were born in the spring to shelters at a time when maternal immunity is waning [29]. However, the season may be a confounding variable, because cats are both seasonal and prolific breeders; an average adult queen can produce two litters between spring and autumn [30].

## Conclusions

The current study provides the first evidence that parvoviruses are circulating among cat populations in Sohag, Assiut, and Cairo provinces. Anorexia, bloody diarrhea, severe dehydration, hypothermia, and vomiting were the most common clinical findings in cats that were positive for parvovirus infection. The geographical location (Sohag) and the season (winter) were statistically significant risk factors associated with parvovirus infection. Our study provides baseline epidemiological data for future management of parvovirus infection and underscores the urgent need for cat vaccination programs based on the current international guidelines.

## Limitations

Although the current study provides precise epidemiological data on parvovirus infection in cats in Egypt, the study population was small, and no molecular characterization was performed to determine whether the current parvovirus infections were caused by FPV or CPV-2. Therefore, genomic surveillance studies involving a large study population from various parts of Egypt are still needed to better shape the epidemiological picture of parvovirus infection in cats.

## Data Availability

The authors declare that the data supporting the findings of this study are available with the paper and confirm that all data generated or anaylsed during this study are included in the publised article.
